# Transcriptome-Wide lncRNA and mRNA Profiling of Spleens from Meishan Pigs at Different Development Stages

**DOI:** 10.3390/ani12192676

**Published:** 2022-10-05

**Authors:** Chao Xu, Jing Shi, Rufeng Huang, Zhengchang Wu, Shenglong Wu, Wenbin Bao

**Affiliations:** 1 Key Laboratory for Animal Genetics, Breeding, Reproduction and Molecular Design of Jiangsu Province, College of Animal Science and Technology, Yangzhou University, Yangzhou 225009, China; 2 Joint International Research Laboratory of Agriculture & Agri-Product Safety, Yangzhou University, Yangzhou 225009, China

**Keywords:** mRNA, lncRNA, Meishan pig, spleen, co-expression, ceRNA, development

## Abstract

**Simple Summary:**

Meishan pig is a local pig breed in China, which has higher immunity than commercial pig breeds for some diseases. The spleen has hematopoietic and immune response functions, making it a good organ model for studying immunity. We depicted the expression profiles of lncRNA-mRNA in the spleen of Meishan pigs at different developmental time points (7 d, 21 d, 35 d, 120 d and 180 d). In addition, we found that AKT3, CBL and PTK2B may be involved in immune regulation in Meishan pigs through a competing endogenous RNA network. This result provides valuable genomic resources for studying immune regulation in animals and finds potential molecular markers for pig disease resistance breeding.

**Abstract:**

Meishan is a well-established local Chinese breed known for its high fecundity, strong immune response and high meat quality. However, the molecular mechanism of immune regulation during the development of Meishan pigs still remains unclear. Here, we performed the transcriptional sequencing of spleen tissues from Meishan pigs at different development stages. In total, 10,268 lncRNAs were identified, including 1254 novel lncRNAs and 9014 known lncRNAs. Time series analysis revealed that genes of the up-regulated module were enriched in pathways associated with transport, immunity, and histone acetylation modifications, while genes of the down-regulated module were enriched in DNA metabolic process and cell cycle. Weighted gene co-expression network analysis (WGCNA) showed the functional linkage between mRNAs and lncRNAs, indicating that lncRNAs are important regulatory elements of mRNAs. Notably, a lncRNA-miRNA-mRNA competing endogenous RNA (ceRNA) network that contained 3 mRNAs (AKT3, CBL and PTK2B), 17 lncRNAs and 67 miRNAs were screened out, which probably plays a critical role in immune regulation of Meishan pigs. Our findings not only revealed the transcriptome profile of spleen development, but also provide novel insights into the mechanism of lncRNA-miRNA-mRNA axis in the immune response in Meishan pigs.

## 1. Introduction

Pigs (*Sus scrofa*) have significant agricultural importance around the world and are increasingly employed as a disease model for humans as well as a source of tissues for xenotransplantation [1,2,3]. The Meishan pig is a Chinese native breed that is famous for its high fertility [4]. As a Chinese local pig breed, Meishan pigs have higher resistance to certain diseases than the commercial pig breeds. It has previously been observed that Meishan pigs are more resistant to enterotoxigenic Escherichia coli K88 than European Large White pigs [5]. Nowak et al. have suggested that Meishan pigs do not display alterations in intestinal permeability in response to lipopolysaccharide (LPS) exposure, whereas Yorkshire pigs demonstrate increased intestinal permeability in response to LPS challenge [6]. Recent evidence also suggests that Meishan piglets have higher intestinal immune function than crossbred piglets [7]. It is widely accepted in China that Meishan pigs have high disease resistance and immunity, but the underlying molecular mechanisms remain unclear. As the spleen is an essential immunological organ, it is crucial to comprehend the mechanism of spleen development in Meishan pigs at different growth stages.

Long noncoding RNAs (lncRNAs) are a heterogeneous type of non-protein-coding transcripts exceeding 200 nucleotides in length [8]. As emerging regulators of gene expression, lncRNAs participate in a variety of physiological and pathological processes. Accumulating evidence suggests that lncRNAs play important functional roles in regulating cell differentiation and tissue development. A previous study has showed that LncRNA Snhg6 modulates the ubiquitination of EZH2 to control the development of Myeloid-derived suppressor cells [9]. Another study has established that LncRNA-Cox2 regulates macrophage polarization and inflammatory responses in septic mice via the CREB-C/EBP signaling pathway [10]. In addition, Qiao et al. [11] have provided transcriptome profiles of mRNAs and lncRNAs at development stages in Yorkshire pigs. However, no studies have explored lncRNA-mRNA interactions during the spleen developmental stages of Meishan pigs. Therefore, we need to explore the spleen lncRNA and mRNA profiles of Meishan pigs at different developmental stages to reveal the potential developmental regulatory mechanisms.

Currently, the spleen serves as a better organ model for studying immunity. In this study, spleen tissues from Meishan pigs at five distinct developmental stages were obtained for lncRNA and mRNA sequencing to uncover molecular networks that regulate immunological interactions during spleen development. The competitive endogenous RNA (ceRNA) hypothesis assumes that LncRNAs can act as adsorption sponges for target miRNAs to regulate mRNA expression levels. By providing a comprehensive view of the transcriptional and regulatory landscape in the developing pig spleen, we demonstrate that the ceRNA interactions network may play an important role in spleen development and help us better understand the regulation of immune development in Meishan pigs.

## 2. Materials and Methods

### 2.1. Animal Collection and Sample Collection

All Meishan piglets were purchased from Kunshan Conservation Ltd. (Suzhou City, Jiangsu Province, China). The Meishan breed is a well-known native Chinese breed renowned for its high fecundity, strong immune system, and high meat quality. In this study, we collected spleens from a total of 5 developmental stages: day 7, day 21, day 35, day 120 and day 180, with 3 healthy male pigs in each stage. Experimental animals were treated using intravenous injection of 2% pentobarbital sodium to minimize pain, followed by rapid dissection of the spleen from each carcass and immediate freezing in liquid nitrogen. Before total RNA extraction, all obtained spleen samples were kept in a freezer at –80 °C.

### 2.2. RNA Extraction and Sequencing Analysis

Following the manufacturer’s instructions, total RNA was isolated using the mirVana miRNA Isolation Kit (Ambion). The Agilent 2100 Bioanalyzer was utilized to assess the integrity of the RNA (Agilent Technologies, Santa Clara, CA, USA). The samples with an RNA Integrity Number (RIN) of less than 7 were used in the following analysis. Following the manufacturer’s instructions, the libraries were created using TruSeq Stranded Total RNA with Ribo-Zero Gold. Afterwards, 150 bp paired-end reads were produced using the Illumina sequencing platform (HiSeqTM 2500 or another platform).

### 2.3. Raw Data Processing and Genomic Alignment

Raw reads generated by high-throughput sequencing are saved in a fastq format file. The raw reads are filtered based on quality values using Trim Galore (https://www.bioinformatics.babraham.ac.uk/projects/trim_galore/, accessed on 5 December 2021). Quality control reports are generated for the filtered clean reads using Fastqc (https://www.bioinformatics.babraham.ac.uk/projects/fastqc/, accessed on 5 December 2021). Quality-qualified clean reads are aligned to the porcine reference genome using hisat2 (version: 2.2.1) [12].

### 2.4. lncRNA Prediction and Gene Quantification

The results of the alignment to the reference genome are saved in a bam file. Reads were assembled using Stringtie software (version: 2.1.5) [13] and subsequently candidate lncRNA transcripts were selected by comparing the gene annotation information generated by Cuffcompare software (version: 2.2.1) [14]. Finally, the transcripts with coding ability were de-filtered by CPC [15], CNCI [16], Pfam [17] and PLEK [18] to obtain the newly predicted lncRANs.

Count value and TPM value were obtained by aligning the reads of each sample using bowtie2 (version: 2.3.5.1) [19] to the mRNA transcripts as well as known and predicted lncRNA sequences.

### 2.5. Differentially Expressed Gene Analysis and Enrichment Analysis

Differential expression gene analysis was performed between the groups (21 d vs. 7 d, 35 d vs. 7 d, 120 d vs. 7 d, 180 d vs. 7 d) using the R package DESeq2 (version: 1.34.0) [20], and genes with p-adj < 0.05 and |log2FC| >1 were selected as differential genes. Functional enrichment analysis of genes based on GO and KEGG databases was performed by hypergeometric distribution test using enrichGO and enrichKEGG in the R package clusterProfiler (version: 4.2.2) [21], and enriched pathways with *p*-values less than 0.05 were retained.

### 2.6. Time-Series Analysis

The fuzzy c-means algorithm provided by the R package Mfuzz (version: 2.54.0) [22] was used to perform soft-clustering analysis to identify different expression patterns of genes in time series experimental designs. For this analysis, two parameters, c (number of clusters) and m (fuzzification parameter), are required. We determined the value of the parameter c by evaluating the sum of squared error between the increasing number of clusters and obtain the value of the parameter m by using the mestimate function in the Mfuzz package. After determining the two main parameters, we performed clustering and ensured that the genetic trends of each group were the same by setting membership >0.8.

### 2.7. Construction of Weighted Gene Co-expression Network

Gene co-expression modules can be created utilizing gene expression profiles by weighted gene co-expression network analysis (WGCNA) [23,24]. The gene relationship matrix was first obtained from the gene expression profile using Pearson correlation coefficient. The results of the gene relationship matrix obtained from Pearson correlation coefficients were converted into a adjacency matrix by setting a soft threshold β of 6. The topological overlap matrix (TOM) was then calculated to measure the interconnectedness of the network. We used the difference degree of TOM as the clustering distance to classify genes into different modules. The dynamic tree algorithm was also used to merge similar gene modules by setting a threshold of 0.25.

### 2.8. Prediction of miRNA Target Gene and CeRNA Network Construction

The miRNA sequences were downloaded from the miRbase (https://mirbase.org/, accessed on 10 August 2021) website and mRNA 3’UTR sequences were obtained from BioMart (https://asia.ensembl.org/index.html, accessed on 5 May 2022) [25]. The 3’UTR sequences of miRNA-targeted mRNAs and lncRNA sequences were then predicted using miRanda (version: 3.3a) [26] software. To improve the accuracy of prediction, we set max score >150 and max energy <–20 as thresholds to screen miRNA-targeted mRNAs and lncRNAs. Finally, ceRNA networks were constructed based on miRNAs shared by mRNAs and lncRNAs. The lncRNA-mRNA co-expression network is visualized using Cytoscape software (version: 3.9.0) [27].

### 2.9. Statistical Analysis and Data Visualization

All statistical analysis is done in R environment (version: 4.1.3) [28] and the visualization of the data is done using the R package ggplot2 (version: 3.3.5) [29].

## 3. Results

### 3.1. The Dynamic Changes of Transcriptional Landscape during Five Different Developmental Stages of the Spleen

**Figure 1:**
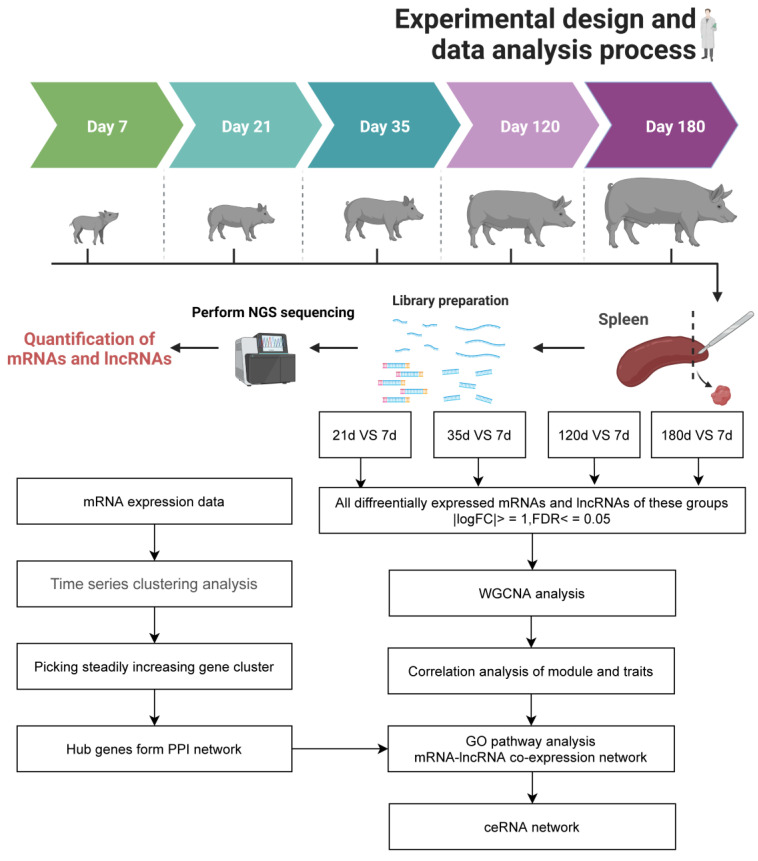
Graphical summary of the experimental design and bioinformatics analysis process. Spleen samples from five different time points (7 d, 21 d, 35 d, 120 d and 180 d) were collected and subjected to RNA-seq (n = 3 samples per time point), followed by downstream analyses.

**Figure 2:**
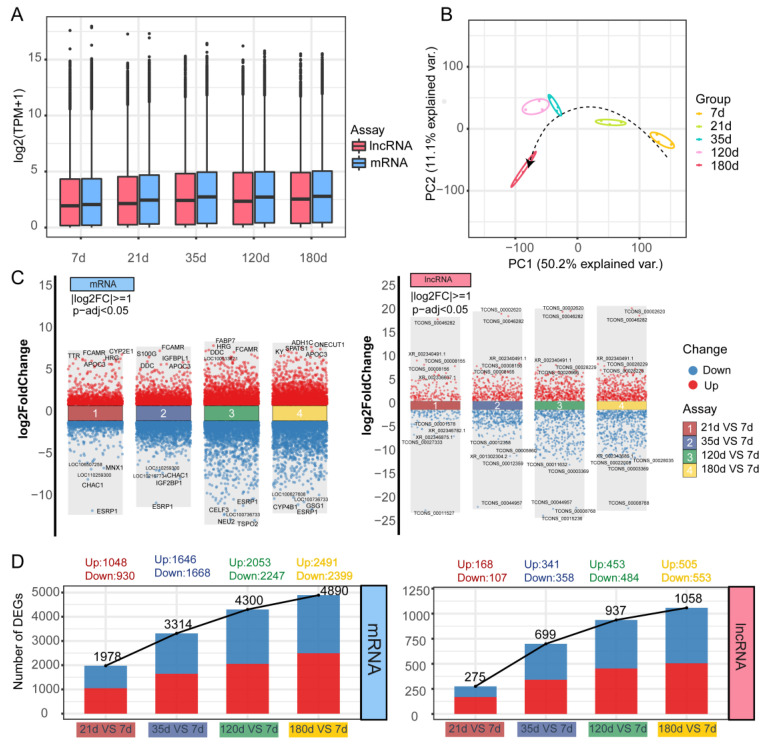
Analysis of differentially expressed genes. (**A**) Box plot of log2(TPM) values for mRNA (pink) and lncRNA (blue) across different time points. (**B**) PCA diagram on normalized mRNA expression values illuminating the general relationship between datasets. The arrows show the developmental trajectory during the different periods. (**C**) Differential gene expression results displaying up- and down-regulated genes in mRNA (left) and lncRNA (right) database between four comparison groups (21 d vs. 7 d, 15 d vs. 7 d, 120 d vs. 7 d, and 180 d vs. 7 d). The threshold for differential expression genes is |log2(FC)| >1 and adjust *p* value <0.05. Blue dots indicate down-regulated genes, and red dots indicate up-regulated genes. The top 5 up- and down-regulated gene symbols are shown in the graph. (**D**) Histogram of the number of differential expression genes for mRNA (left) and lncRNA (right) database.

The experimental design and data analysis process is depicted in Figure 1. The first set of analysis aimed to demonstrate differences at the transcriptome level at five different developmental time points. The boxplot distribution of TPM shows the median and quartile values of mRNA and lncRNA expression among different time points. (Figure 2A and Tables S1 and S2). Principal component analysis revealed that the different groups may be distinguished from one another with relative ease, ranked according to their day of the sample (Figure 2B). Furthermore, Differential gene expression analysis was conducted, and great changes were shown at day 21, day 35 and day 120 compared to day 7 (Figure 2C). More specifically, differentially expressed genes (DEGs) showed an upward trend in the different comparison groups, with 1978 mRNA and 275 lncRNA in the comparison group day 21 vs. day 7, 3314 mRNA and 699 lncRNA in the group day 35 vs. day 7, 4300 mRNA and 937 lncRNA in the group day 120 vs. day 7, 4890 mRNA and 1058 lncRNA in the group day 180 vs. day 7 (Figure 2d). In order to assess the biological processes of up-regulated differential genes and down-regulated differential genes in the different comparison groups, gene set enrichment analyses were used (Tables S3–S10). Gene ontology (GO) characteristics related to innate immune response were detected across up-regulated differential genes, whereas the down-regulated differential genes were enriched in cell cycle and DNA replication (Figure S1A). KEGG pathway enrichment analysis suggested that cell differentiation, DNA replication and cell cycle are co-enriched in DEGs in different comparison groups to the pathway.

### 3.2. Temporary and Continuously Changing Transcriptional Programs in the Spleen Development

**Figure 3:**
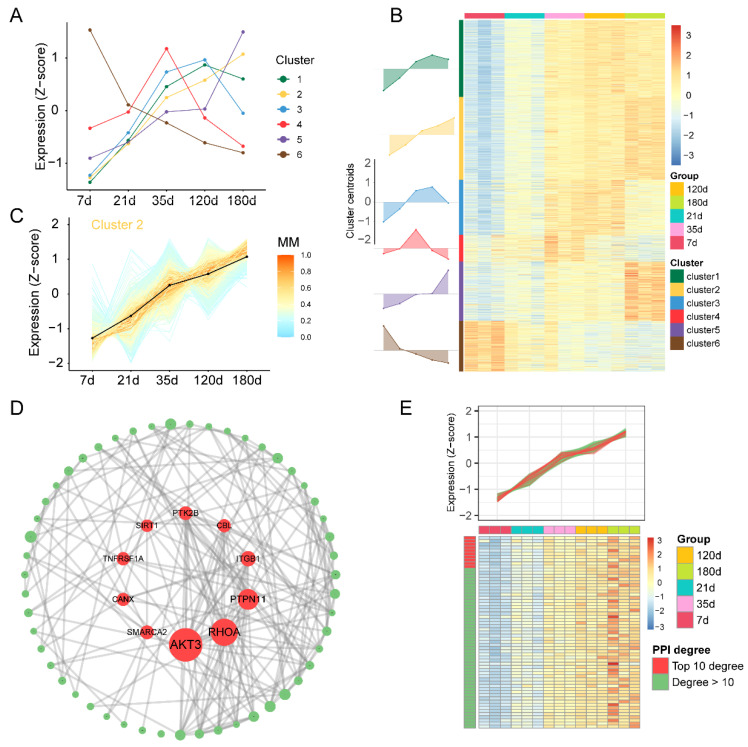
Global changes in gene expression across multiple time points and identification of hub genes. (**A**) Line plot displaying the expression patterns of mRNAs and cluster centroids identified by the fuzzy c-means algorithm at different developmental time points. (**B**) Heatmap displaying six obtained clusters with dynamic gene expression patterns. The clusters’ overall gene expression dynamics are displayed in area plots (on the left) (visualized in relation to cluster centroids). (**C**) Line plot displaying the dynamics of all genes (expression Z-score) within cluster 2. Black lines are used to depict centroids. The correlation between a specific gene and its centroid is displayed by color density. (**D**) The protein-protein interaction (PPI) network constructed using the STRING database for genes in cluster 2 (membership > 0.8). The size of each node in the network indicates its degree, and nodes with degrees greater than 10 are displayed. The 10 red nodes in the middle of the network are the hub genes. (**E**) Heatmap showing the expression pattern of genes in the PPI network.

Next, we aimed to describe broad patterns and shifts among the transcriptional changes using clustering (Table S11). Based on their scaled and centered average expression values, the fuzzy c-means clustering algorithm was applied to protein-coding genes (Figure 3A), using calculated optimal cluster number (k = 6) via gap statistics (Figure S2A). Heatmap (Figure 3B) and line plots (Figure 3C; and Figure S2B–F) show the dynamically changing transcriptomic profile in spleen development. Among the six clusters, we can distinguish transcriptional programs with unsteady (clusters 1, 3, 4, and 5) or steady changing (clusters 2 and 6) dynamics (Figure 3C; and Figure S2E). Clusters 1 (green) and 3 (blue) contain 2778 and 1984 protein-coding genes, respectively, which have a low expression level at day 7, steadily rise, and then begin to decline at day 120 (Figures S2B and S2C). The difference is that cluster 5 (purple) contains 2140 genes that rise at the very beginning but remain flat from 120 to 180 days and then start rising again (Figure S2E). Cluster 4 (red) contains 970 genes that up-regulated before day 35 but then down-regulated (Figure S2D). Cluster 6 (brown) contains 1822 genes, which have been in a decreasing expression pattern (Figure S2E). We found that the gene expression in cluster 2 increased with time (Figure 3b). Therefore, we chose to concentrate on cluster 2 (yellow), which comprises 2982 protein-coding genes and has a steadily growing pattern of gene expression (Figure 3C). Categories associated with biological processes were found using gene ontology (GO) analysis (Table S12). Specifically in cluster 2, we observed an enrichment in pathways such as those associated with Golgi vesicle transport, vesicle-mediated transport, histone H4 acetylation, i.e., (Figure S2G). In the next step, the genes with a membership score greater than 0.8 were used for protein interaction network analysis, and the nodes with a degree greater than 10 were shown. The genes with rank in the top 10 according to degree were used as hub genes (AKT3, RHOA, PTPN1, ITGB1, CBL, PTK2B, SIRT1, TNFRSF1A, CANX, and SMARCA2; Figure 3D and Table S13). The heatmap shows a steady upward expression pattern of the hub genes at different time points (Figure 3E). Collectively, our findings provide information on possible genes involved in the development of the pig spleen.

### 3.3. Construction of mRNA-lncRNA Co-expression Networks using WGCNA

**Figure 4:**
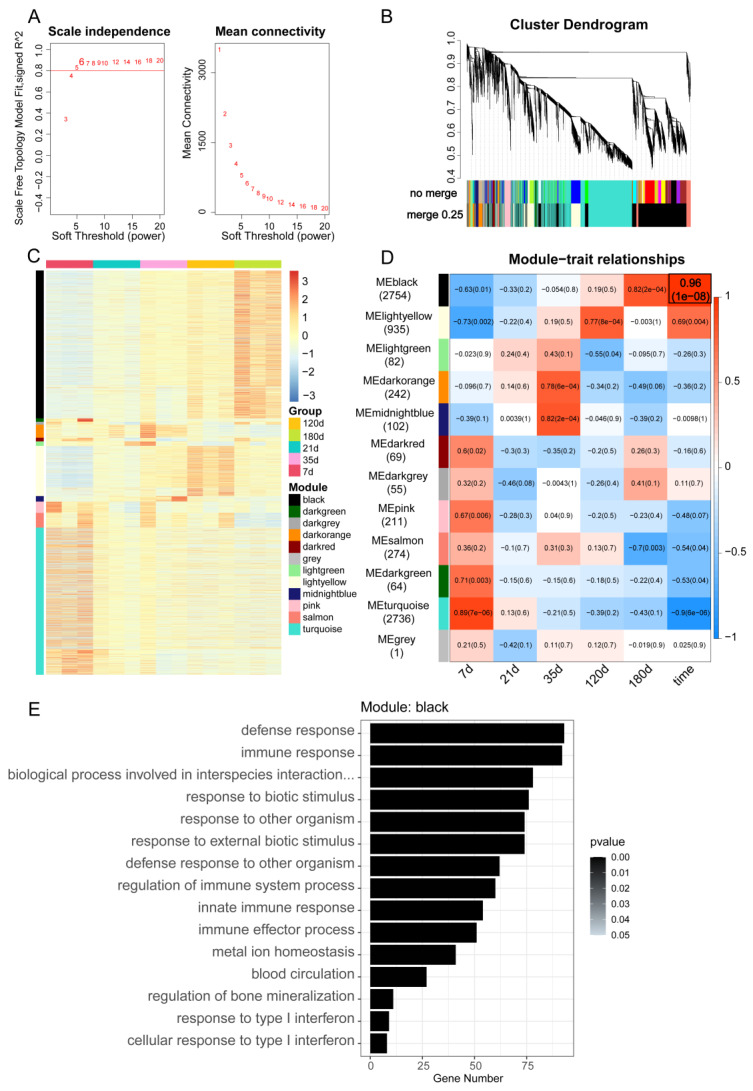
WGCNA of mRNA and lncRNA dataset identified gene co-expression module at different time points. (**A**) Selection of the soft-thresholding powers (β). The scale-free fit index in relation to soft-thresholding power was displayed in the left panel. The mean connectivity vs soft-thresholding power was shown in the right panel. The fit index curve for power 6 was chosen because it flattens out at high value (>0.8). (**B**) Hierarchical cluster dendrogram of spleen samples in different time points showing co-expression modules generated using WGCNA. Modules belonging to branches are color-coded according to the interconnectedness of genes. 12 modules represented by colors in the horizontal bar were found using 0.25 threshold merging. (**C**) Heatmap showing gene expression of 12 modules across five time points. (**D**) Relationship between modules and traits. Each column denotes a trait, and each row denotes an eigengene for a certain module. The matching correlation and *p* value are included in each cell. (**E**) GO enrichment analysis of mRNAs from the black module. The bar chart displays the top 15 highly enriched biological processes, ordered by *p*-value.

All DEGs from Figure 1 were used to construct co-expressed gene modules using WGCNA. Thereafter, The heterogeneity of each sample was examined using hierarchical clustering analysis in order to find and eliminate outliers (Figure S3A). To better match the scale-free network and have more biological significance, the soft threshold power β was chosen as 6. When the power value was equal to 6, the independence was very high, and the average connectedness was relatively low (Figure 3A). Therefore, β = 6 was used to construct a gene hierarchy clustering tree and 12 gene modules (black, darkgreen, darkgrey, darkorange, darkred, grey, lightgreen, lightyellow, midnightblue, pink, salmon, turquoise) were identified by average link clustering to place mRNAs and lncRNAs with the same expression pattern into the same modules (Figure 4B and Table S14). The heatmap shows the expression pattern of different modules, where the expression of genes in the black module tends to increase with time (Figure 4C). Furthermore, analysis of the module–trait relationship showed that different developmental time points in the spleen were significantly and positively correlated with the black module (Figure 4D). We hypothesize that genes in the black module are involved in the developmental process of the spleen in a deterministic manner and can reveal the mRNA–lncRNA interaction events associated with this biological process. Subsequently, we performed functional annotation analysis of the gene set based on the GO database for the genes within the different modules separately (Figure 4E, Figure S3B–J and Table S15). The results show that the genes within the black module are involved in biological processes mostly related to immunity, such as defense response, immune response, and innate immune response i.e., (Figure 4E). Taken together, these findings suggest that we have constructed mRNA-lncRNA co-expression modules with different expression patterns and that the black module is significantly positively correlated with the time trait, which may have an essential role in spleen development.

### 3.4. Construction of CeRNA Network through lncRNA-mRNA Co-expression Relationship

**Figure 5:**
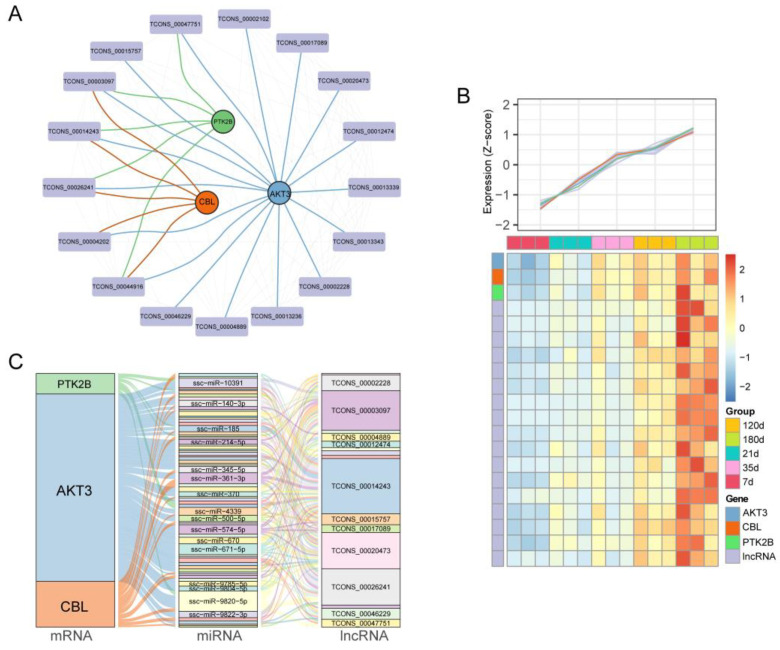
Co-expression network and ceRNA regulation mechanism. (**A**) Co-expression mRNA-lncRNA network of ATK3 (blue), PTK2B (green) and CBL (orange) with its co-expression lncRNAs (purple) in black module. The circular nodes represent the mRNAs, square nodes represent lncRNAs. (**B**) Heatmap showing the trend of gene expression in the co-expression network. (**C**) Sankey plot for the ceRNA network predicted by the co-expression network. The size of each rectangle, which stands for a gene, displays the degree to which that gene is connected to others.

Next, we found that three of the ten core genes from Figure 3 were in the black mRNA-lncRNA co-expression module, and 17 lncRNAs were co-expressed with it (Figure 5A and Table S16). Heatmap shows the expression pattern of AKT3, CBL, PTK2B and their co-expressed lncRNAs with an increasing trend over time (Figure 5B). It is generally known that lncRNAs and mRNAs exhibit co-expression patterns in ceRNA networks. To clarify how lncRNAs regulate mRNAs expression by competitively binding miRNAs as adsorption sponges, we construct the ceRNA network. This ceRNA interaction network contains a total of three mRNAs (AKT3, CBL, PTK2B), 67 miRNAs (ssc-miR-10391, ssc-miR-140-3p, ssc-miR-185, ssc-miR-345-5p, ssc-miR-361-3p, ssc-miR-4339, ssc-miR-500-5p, ssc-miR-574-5p, ssc-miR-670, ssc-miR-671-5p, ssc-miR-9820-5p, ssc-miR-9822-3p, etc.) and 17 lncRNAs (TCONS_00003097, TCONS_00014243, TCONS_00026241, TCONS_00047751, TCONS_00002102, TCONS_00002228, TCONS_00004889, TCONS_00012474, TCONS_00013236, etc.) (Figure 5C). In summary, we obtained ceRNA interaction networks for three hub genes that may potentially be important for spleen development in pigs.

## 4. Discussion

As a local pig breed in China, Meishan pigs are famous for their high immunity. Several previous studies have shown that Meishan pigs have stronger immunity and higher resistance to certain diseases than commercial breed pigs. Recently, splenic transcriptome profiles of commercial Yorkshire pigs have been provided at different developmental stages to investigate potential immune regulatory mechanisms. However, the immune regulatory mechanisms of the spleen in Meishan pigs at different developmental periods are still unclear. In this study, we selected spleen tissues from Meishan pigs at 7 days, 21 days, 35 days, 120 days and 180 days for transcriptome sequencing and the findings provide valuable resources for mRNA-lncRNA profile to further investigate the immunological properties of Meishan pigs.

In this study, we found 10,268 lncRNAs in spleen tissues of 15 Meishan pigs, including 1254 novel lncRNAs and 9014 known lncRNAs. Using 7 days as the control group, differential analysis revealed that the number of differential lncRNAs and differential mRNAs both increased with increasing age, indicating a regulatory relationship between lncRNAs and mRNAs. Subsequently, we found six modules with different gene expression patterns by time series analysis, in which genes in the up-regulated module were enriched in GO biological pathways with histone H4 acetylation, Golgi vesicle transport, immune effector transport process and defense response, whereas genes in the down-regulated module were enriched in the cell cycle and DNA metabolic process. This dynamically changing gene expression pattern can well reflect the biological functions of the spleen. In addition, we found 10 hub genes (AKT3, RHOA, PTPN1, ITGB1, CBL, PTK2B, SIRT1, TNFRSF1A, CANX, and SMARCA2) whose expression levels increased with increasing age. The hub genes identified in this study may serve as valuable candidates for further understanding the mechanisms of immune regulation during spleen development. According to several studies [30,31,32], AKT3 (AKT serine/threonine kinase 3) has been associated with metabolism, growth, and differentiation. In addition, mutations in AKT3 may also lead to developmental disorders [33,34]. RHOA (ras homolog family member A), a member of the Rho kinase family, was involved in the growth and migration of B- and T-cells [35]. A previous study had revealed that knockdown of RHOA significantly hampers B cell development within the mouse spleen, resulting in a significant reduction in the number of marginal zone, transitional and follicular B cells [36]. PTPN1 (Protein tyrosine phosphatase 1B, also known as PTP1B) was an essential regulator of signaling pathways that regulate metabolic balance, cell proliferation, and immunity [37]. Reth et al. [38] showed that PTPN1 adversely regulates CD40, B cell-activating factor receptor, and TLR4 signaling in B cells. ITGB (Integrin-β) was a member of the integrin superfamily and was essential for hemostasis, tissue repair and immune response [39]. As an upstream molecule of the Wnt/β-catenin signaling pathway, ITGB1 played a crucial role in immune suppression in gastric cancer [40]. CBL (Cbl proto-oncogene B) proteins had multiple roles in regulating signal transduction, and their absence can mentor malignancy and immune disorders [41]. PTK2B (protein tyrosine kinase 2 beta) had recently been found to modulate inflammatory responses through the IRF5 innate immune sensing pathway in the gut [42]. SIRT1 (sirtuin 1) had been reported to participate in the regulation of inflammation, oxidative stress, mitochondrial function, immunological responses, cellular differentiation, proliferation, and metabolism [42]. TNFRSF1A (TNF receptor superfamily member 1A, also known as TNFR1) was an important regulator of cell proliferation and death and regulates the NFκB pathway through the ubiquitin/proteasome system [43]. CANX (calnexin) was viewed as an important member of the antigen processing pathway and acts as a chaperone protein involved in the folding and assembly of MHC-I molecules on the endoplasmic reticulum [44,45]. SMARCA2, also known as Brahma homologue (BRM), was essential for cell proliferation, linage specification and development, cell adhesion, cytokine responses, and DNA repair [46].

lncRNAs may control physiological and pathological changes, exhibit excellent spatiotemporal specificity during tissue development, and influence gene expression at the epigenetic level, transcriptional and post-transcriptional level [47,48,49,50]. We constructed different co-expression network modules of mRNA-lncRNAs associated with different time points by WGCNA and found that genes within the black module were significantly enriched in biological pathways related to the immune response, suggesting that the black module may play an important role in immune regulation in spleen development. According to the ceRNA hypothesis, lncRNAs act as adsorption sponges for miRNAs to indirectly regulate mRNA expression levels [51,52]. Recently, several studies have implicated ceRNA in tissue development and immune regulation [53,54,55]. According to our results, we successfully constructed the lncRNA-miRNA-mRNA ceRNA network, which shows that it may have an important role in regulating spleen development. According to our results, we successfully constructed a lncRNA-miRNA-mRNA ceRNA network containing three mRNAs (AKT3, CBL, PTK2B), 17 lncRNAs (TCONS_00003097, TCONS_00014243, TCONS_00026241, TCONS_00047751, TCONS_00002102, TCONS_00002228, TCONS_00004889, TCONS_00012474, TCONS_00013236, etc.) and 67 miRNAs (ssc-miR-10391, ssc-miR-140-3p, ssc-miR-185, ssc-miR-345-5p, ssc-miR-361-3p, ssc-miR-4339, ssc-miR-500-5p, ssc-miR-574-5p, ssc-miR-670, ssc-miR-671-5p, ssc-miR-9820-5p, ssc-miR-9822-3p, etc.). Among these miRNAs, a number of studies have already demonstrated their potential involvement in tissue development and immunological function. For example, a study showed that mir-423-5p may be an important regulatory molecule for testis development and fertilization [56]. miR-185 was found to promote proliferation of intestinal epithelial cells [57]. miR-10391 had been reported to regulate the process of swine influenza virus infestation of host cells through ceRNA mechanism [58].

## 5. Conclusions

In conclusion, this study provided the first transcriptome profiles of mRNA and lncRNA in Meishan pig spleen tissue at different time points. It was speculated that 10 mRNAs (AKT3, RHOA, PTPN1, ITGB1, CBL, PTK2B, SIRT1, TNFRSF1A, CANX, and SMARCA2) are associated with immune regulation during spleen development. Our results also contributed a ceRNA network of lncRNA-miRNA-mRNA and suggest that lncRNAs may have multiple roles in the regulation of spleen development. Several limitations still remained in this study, and we need to validate our candidate molecules through experiments further.

## Data Availability

The RNA-seq data from this study are available in GenBank Sequence Read Archive (SRA) database with accession number PRJNA875155.

## Supplementary Materials

The following supporting information can be downloaded at: https://www.mdpi.com/article/10.3390/ani12192676/s1, Figure S1: RNA-seq soft clustering for Splenic transcriptional landscape, Figure S2: GO enrichment analysis of genes within co-expression modules, Table S1: Differentially expressed mRNAs in pairwise stages, Table S2: Differentially expressed lncRNAs in pairwise stages, Table S3: GO functional categories of mRNAs differentially expressed in 21d vs 7d, Table S4: GO functional categories of mRNAs differentially expressed in 35d vs 7d, Table S5: GO functional categories of mRNAs differentially expressed in 120d vs 7d, Table S6: GO functional categories of mRNAs differentially expressed in 180d vs 7d, Table S7: KEGG pathways of mRNAs differentially expressed in 21d vs 7d, Table S8: KEGG pathways of mRNAs differentially expressed in 35d vs 7d, Table S9: KEGG pathways of mRNAs differentially expressed in 120d vs 7d, Table S10: KEGG pathways of mRNAs differentially expressed in 180d vs 7d, Table S11: mRNA list in different module profiles of time series analysis, Table S12: GO functional categories of mRNAs form different module profiles of time series analysis, Table S13: PPI network, Table S14: mRNA and lncRNA list in co-expression network, Table S15: GO functional categories of mRNAs form different co-expression network, Table S16: ceRNA network.
