# Supplementary material for: Transcriptome-Wide lncRNA and mRNA Profiling of Spleens from Meishan Pigs at Different Development Stages
Source: Animals (Basel). 2022 Oct 5;12(19):2676. doi: 10.3390/ani12192676 (PMC9558508; doi:10.3390/ani12192676)
Supplement: Supplementary file 1 [file animals-12-02676-s001.zip › animals-1918231-supplementary/Supplementary Materials/Supplementary Figures.pdf]

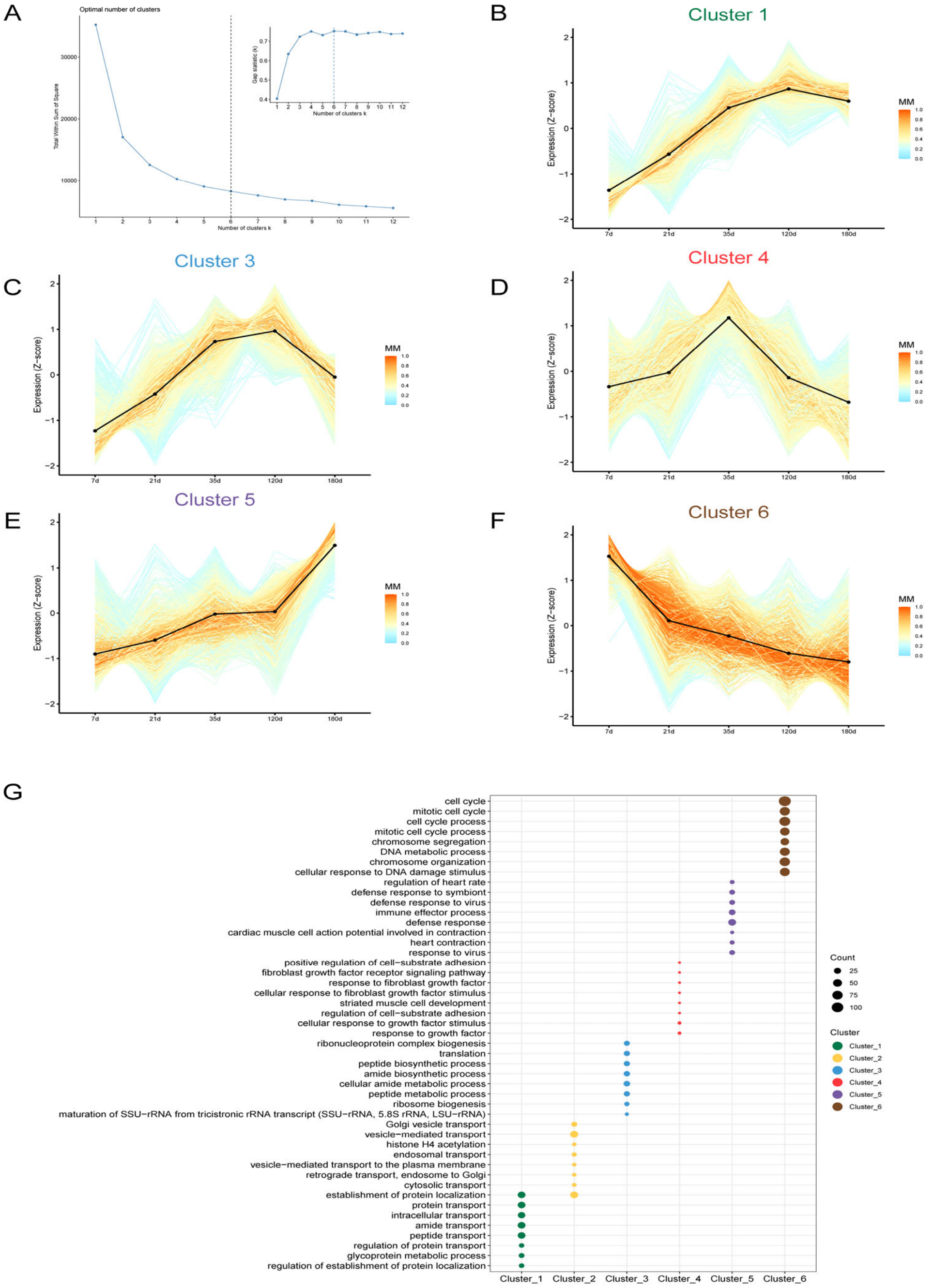

Figure S1. RNA-seq soft clustering for Splenic transcriptional landscape. A. Elbow-plot of the sum of squares with increasing number of clusters to determine the optimal number of clusters in the RNA-seq datasets from 7d to 180d. The black dashed line shows the ideal number of clusters (k = 6). The outcomes of applying the "gap statistic" method are shown in the inset by comparing the change in within-cluster dispersion with that anticipated under a suitable reference null distribution (the blue dashed line represents the ideal number of clusters, k = 6). **B-F.** Line plot displaying the expression patterns of each gene at every cluster. Black lines are used to depict centroids. The association between a specific gene and its centroid is represented by color density. **G.** GO enrichment analysis of genes within each cluster. Different colors represent different clusters, and the size of the dots represents the number of genes.

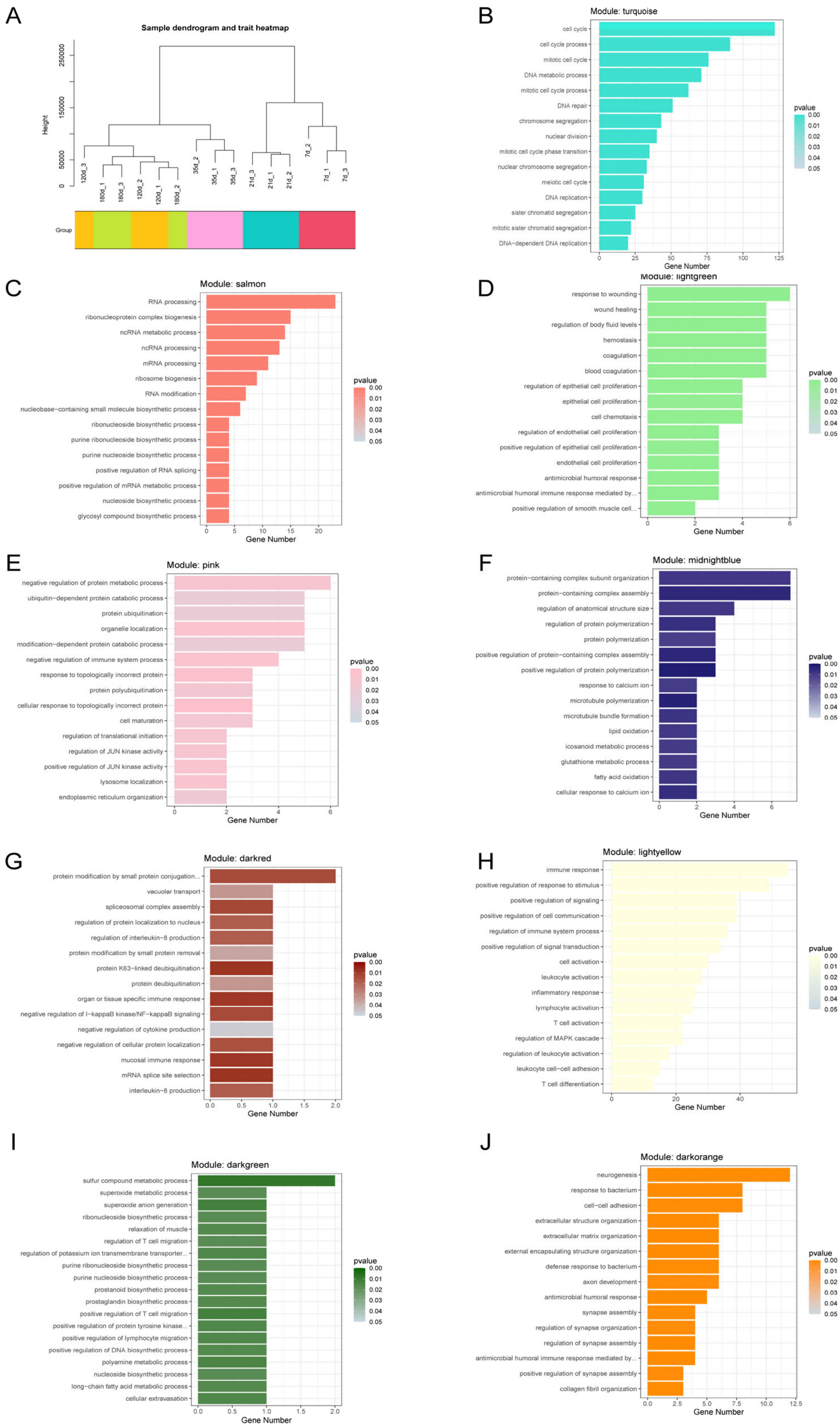

Figure S2. GO enrichment analysis of genes within co-expression modules. A. The cluster dendrogram of the spleen sample at different developmental time points. **B-J.** Bar plot showing the results of the GO enrichment analysis.
